# An Improved Cloud Classification Algorithm for China’s FY-2C Multi-Channel Images Using Artificial Neural Network

**DOI:** 10.3390/s90705558

**Published:** 2009-07-14

**Authors:** Yu Liu, Jun Xia, Chun-Xiang Shi, Yang Hong

**Affiliations:** 1 Key Laboratory of Water Cycle and Related Land Surface Processes, Institute of Geographic Sciences and Natural Resources Research, Chinese Academy of Sciences, Beijing, 100101, China; E-Mail: liuyu950@126.com; 2 Graduate School of the Chinese Academy of Science, CAS, Beijing, 100039, China; 3 National Satellite Meteorological Center, Beijing 100081, China; E-Mail: shicx@cma.gov.cm; 4 School of Civil Engineering and Environmental Sciences, University of Oklahoma, National Weather Center Suite 3630, Norman, OK 73019, USA; E-Mail: yanghong@ou.edu

**Keywords:** FY-2C, multi-channel satellite image, ANN, cloud classification

## Abstract

The crowning objective of this research was to identify a better cloud classification method to upgrade the current window-based clustering algorithm used operationally for China’s first operational geostationary meteorological satellite FengYun-2C (FY-2C) data. First, the capabilities of six widely-used Artificial Neural Network (ANN) methods are analyzed, together with the comparison of two other methods: Principal Component Analysis (PCA) and a Support Vector Machine (SVM), using 2864 cloud samples manually collected by meteorologists in June, July, and August in 2007 from three FY-2C channel (IR1, 10.3–11.3 μm; IR2, 11.5–12.5 μm and WV 6.3–7.6 μm) imagery. The result shows that: (1) ANN approaches, in general, outperformed the PCA and the SVM given sufficient training samples and (2) among the six ANN networks, higher cloud classification accuracy was obtained with the Self-Organizing Map (SOM) and Probabilistic Neural Network (PNN). Second, to compare the ANN methods to the present FY-2C operational algorithm, this study implemented SOM, one of the best ANN network identified from this study, as an automated cloud classification system for the FY-2C multi-channel data. It shows that SOM method has improved the results greatly not only in pixel-level accuracy but also in cloud patch-level classification by more accurately identifying cloud types such as cumulonimbus, cirrus and clouds in high latitude. Findings of this study suggest that the ANN-based classifiers, in particular the SOM, can be potentially used as an improved Automated Cloud Classification Algorithm to upgrade the current window-based clustering method for the FY-2C operational products.

## 1. Introduction

Clouds play an important role in the Earth system. They significantly affect the heat budget by reflecting short-wave radiation [1], and absorbing and emitting long-wave radiation [2]. The net effect is a function of the cloud optical properties and the properties of the underlying surface [3]. Different types of clouds have different radiative effects on the Earth surface-atmosphere system. Accurate and automatic cloud detection and classification are useful for numerous climatic, hydrologic and atmospheric applications [4]. Therefore, an accurate and cost-effective method of cloud detection and classification based on satellite images has been a great interest of many scientists [5,6].

Cloud classification methods can mainly be divided into following categories: the threshold approach, traditional statistical methods and new methods such as Artificial Neural Network (ANN). The threshold methods were mainly developed during the 1980s and early 1990s. They apply a set of thresholds (both static and dynamic) of reflectance, brightness temperature and brightness temperature difference [7,8]. They are the simplest and probably most commonly used methods. However, these methods may fail when two different classes have no obvious brightness temperature difference (i.e., indistinct threshold) because of the complexity of the cloud system. The traditional statistical methods, such as clustering method, histogram approach and others [9–11] are supposed to be superior to the threshold methods to conduct cloud classification and detection in that they digest more information by using all the available bands but they can hardly separate clusters with significant overlapping spaces.

With a rapid development in technological innovations, at present, some new methods, such as neural network [12], Bayesian methods [13], maximum likelihood [14] and fuzzy logic [6], have provided impressive results for cloud detection and classification. Many studies have acknowledged that the well-trained cloud classification neural networks usually have relatively superior performance [15,16]. In fact, almost all the classification methods in the first two categories can be seen as a special or simpler case of neural networks [17]. Therefore, since the first application of ANN in cloud classification [12], lots of ANN methods have been applied to satellite infrared images. For example MLP (Multilayer Perceptron) on LandSAT [18] and on NOAA-AVHRR [19], PNN (Probabilistic Neural Network) on GOES-8 and AVHRR [20,21], the combination of PNN and SOM (Self-Organizing Map) on Meteosat-7 [22], RBF (Radial Basis Functions) on GMS-5 [23,24] and so on. However, there is inadequate study to evaluate the performance and capacity of these ANN classifiers on multi-channels satellite imagery. Historically, due to diversity of cloud dynamics and complexity of underlying surface, it is not uncommon to find out that single Infrared channel data could not effectively identify cloud types because different cloud types might have similar cloud-top brightness temperatures (Tbb).

The intention of this study was to evaluate the performance of several widely used classification algorithms (ANN and statistical classifiers) on three data channels (IR1, 10.3–11.3 μm; IR2, 11.5–12.5 μm and WV 6.3–7.6 μm) from China’s first operational geostationary meteorological satellite FemgYu-2C (FY-2C). FY-2C was launched successfully from Xichang city, Sichuan Province, China on October 19, 2004. The present operational cloud classification product of FY-2C is based on a clustering method and uses 32 × 32 pixels window as basic classification unit. This method uses single Infrared channel (IR1) data to detect clouds, then use Tbb gradient of WV channel to classify high-level clouds. It provides unrealistic cloud edges at two adjacent windows due to its large classification unit (32 × 32 pixels). It has limited capacity in identifying low-level cloud and thin cirrus from underlying surface, because it does not make use of FY-2C multi-Infrared split window information which is proved to be useful in cloud detection.

At present, no other more sophisticated cloud classification methods are used in FY-2C operational products. Therefore one overarching goal of this study is to identify more suitable techniques for the multi-channel cloud image classification in order to upgrade the currently use of FY-2C. Results of this study will also help choosing automated cloud classification algorithms for the upcoming launch of the FY-4 series [25]. This paper is organized as the follows. Section 2 introduces the FY-2C images and data. Section 3 provides a brief description of the classification methods. In Section 4, the capability of ANNs is demonstrated and compared with two other traditional classification methods and the current FY-2C operational classification method at two levels: pixel level and image level. The discussions and summary are given in Section 5.

## 2. Data

### 2.1. Satellite Data

FY-2C is positioned over the equator 105° E, and carries VISSR (Visible and Infrared Spin Scan Radiometer). Its nadir spatial resolution is 1.25 km for visible channel, and 5 km for infrared channels (Table 1).

According to the remote sensing characteristics, FY-2C split window (IR1, IR2) can discriminate underlying surface and cloud area. The water vapor channel (WV) can indicate the height of clouds well. VIS is useful for the detection of low clouds, but it is not accessible at night. IR4 is sensitive to objects with higher temperature. It is usually used for the estimation of underlying surface temperature and detection of fog and low-level clouds. However, great efforts are needed to eliminate the influence of visible light on the Tbb of IR4 channel [26]. To develop an automatic cloud classification system by which clouds in daytime and night can be compared, this study chooses three infrared channels data: IR1, 10.3–11.3 μm; IR2, 11.5–12.5 μm; WV 6.3–7.6 μm.

### 2.2. Classes

Our goal was to design and build a proper cloud classification system that is less dependent on regions with strong sunlit conditions and surfaces that are covered by snow or ice. For a classifier to be effective, one must first define a set of classes that are well separated by a set of features derived from the multi-spectral channel radiometric data. The choice of classes is not always straightforward and may depend upon the desired applications. For instance, some investigators choose a set of standard cloud types such as cirrostratus, altocumulus, or cumulus [21,27,28] to show weather condition and rainfall intensity.

The present FY-2C operational cloud classification method divides cloud/surface into seven categories: sea, stratocumulus& altocumulus, mixed cloud, altostratus& nimbostratus, cirrostratus, thick cirrus and cumulonimbus. Because of the influence of FY-2C resolution, it is difficult to identify altostratus and altocumulus. Therefore this study categorized both of them as midlevel clouds. Considering significant differences between thin cirrus and thick cirrus clouds and their impacts on solar radiation, this study also breaks down cirrus clouds into thick and thin one. In addition, with richly educated and trained experience, it is possible for meteorology experts to identify stratocumulus (which is the main form of low-level clouds during this study period) from altocumulus based on brightness temperature and cloud texture. The set of classes used in this study are shown in Table 2.

### 2.3. Samples

According to numerous studies, trained meteorologists rely mainly on six criteria in visual interpretation of cloud images: brightness, texture, size, shape, organization and shadow effects. In this study we invited Dr. Chun-xiang Shi and Professor Xu-kang Xiang to act as experienced meteorologists. Both of them have worked on analysis of satellite cloud images for over 20 years at the National Satellite Meteorological Center of China. They have developed the cloud classification system of NOAA-AVHRR and GMS 4 in China [22]. Therefore, we treat pixel samples collected by meteorologists as the “truth”. The sample collection process can be described by the following steps:

1. *Pre-processing:* Download FY-2C level 1 data of June, July and August in 2007 in HDF format. Then prepare underlying surface map and the Tbb map of three infrared channels (IR1, 10.3–11.3 μm; IR2, 11.5–12.5 μm and WV 6.3–7.6 μm).
2. *Data visualization:* According to its time stamp order, open FY-2C Tbb maps of three infrared channels and underlying surface map at the same time with special human-computer interactive software. The software is developed by Dr. Cang-Jun Yang in NSMC (National Satellite Meteorological Center in Beijing) in the Window PC environment.
3. *Pixel Sample collection:* Scan image and find out a cloud patch whose cloud type is desired, such as cumulonimbus (Cb), thick cirrus according to the experience of our invited meteorological experts. Then choose one pixel at the center of the cloud patch and record its related information: Tbb of IR1, IR2, and WV. This method only chooses one pixel in one cloud patch, and it discards indecipherable cloud patches even with expert’s eyes. Therefore, the samples collected in this study are clearly defined typical cloud types and can be deemed as “truth”. Repeat the sample pixel collection process for the whole image.
4. *Sample Database establishment: Repeat step 2 and 3.* In this study, we collect about 15 pixel samples at one timestamp from the multi-channel images. There are about 200-timestamp multi-channel images have used and 2864 samples of cloud types have been collected. These samples covered almost all types of the geographical regions which are spread over mountains, plains, lakes, and coastal areas. These samples were collected during different period of the day to account the diurnal features of clouds. The number of sample pixels for each category of surface/clouds is shown in Table 2.

### 2.4. Features

Feature extraction is an important stage for any pattern recognition task especially for cloud classification, since clouds are highly variable. We have collected about 34 features on cloud spectral, gray, texture, size features and so on. In order to reduce the dimensionality of the data and extract the features for cloud classification, this study chooses the widely used gray level co-occurrence matrices (GLCM) method. For this approach, a total of 15 feature values were extracted which grouped into three categories (Table 3): gray features of 3 channels (IR1, IR2 and WV), spectral features of 3 channels, and 9 assemblage features of gray features and spectral features. Spectral features are values of either Tb or reflectance, and the gray features are the transformation of Tb/reflectance to [0 255].

### 2.5. Reasonableness Test of Samples

According to statistical theory, the sample probability distribution is assumed to help us to remove some apparent unreasonable data such as outliers, and to understand cloud features. For example, split window channel can identify cloud from non-cloud area if the Tbb value of IR1-IR2 (T1–T2) is less than 0 [22].

**Figure 1.**
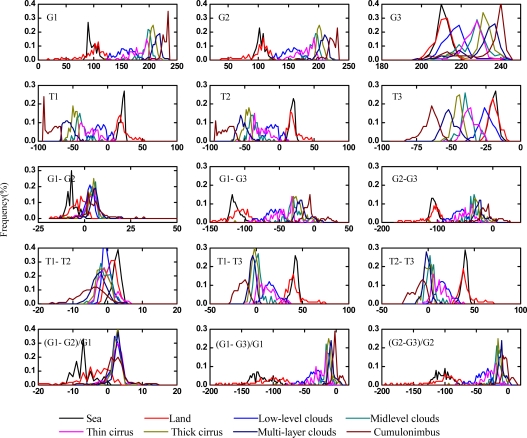
The frequency distribution of features of FY-2C cloud samples.

As shown in Figure 1, samples obey to normal distribution well and the Tbb values of IR1-IR2 (T1–T2) of 98% samples are less than 0. It shows that samples collected in this study are reasonable. From Figure 1, it is common to find that some cloud probability lines are overlapped because of the complexity of the cloud itself. For example, different types of clouds may evolve from or to each other and Tbb of same kind cloud may vary greatly from different region and time. To solve this problem, this study tried collecting as many typical samples as possible to account for all the variations.

### 2.6. Configuration

**Figure 2.**
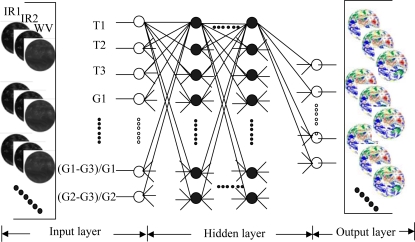
Configuration for the cloud classification: on the lefts are the input satellite images; at the middle are features extracted by GLCM and configuration of classifier; and on the rights are the output cloud classification results. Note white circles on the left are input neurons, and in the right are output ones. Black circles are neurons in hidden layer. Lines around circles show the data flow.

Based on the information mentioned before, the proposed cloud classifiers structure used in this study is as shown in Figure 2.

## 3. Methodology

### 3.1. Cloud Classifier

ANN is a biologically inspired computer program designed to simulate the way in which the human brain processes information. It is a promising modeling technique, especially for data sets having non-linear relationships, which are frequently encountered in cloud classification processes. ANN is usually made up of three parts: input layer, output layer and several hidden layers. Each layer contains number of neurons. Each neuron receives inputs from neurons in previous layers or external sources and then converts inputs either to an output signal or to another input signal to be used by neurons in the next layer. Connections between neurons in successive layers are assigned weights, which represent the importance of that connection in the network. More information on ANN can be found in Reed and Marks [29].

Among the dozens of neural networks available to date, for the approaches to model training they can be divided into two types, according to the need for training samples: supervised ones and unsupervised ones. The former need the user to provide sample classes. They are good at prediction and classification tasks. The latter are input data dictated to find relationships in complex systems. In order to identify what kind of neural networks works best for the FY-2C cloud classification system, the paper compared six of the most frequently used neural networks: Back Propagation(BP), Probabilistic Neural Network (PNN), Modular Neural Networks(MNN), Jordan-Elman network, Self-Organizing Map (SOM), and Co-Active Neuro-Fuzzy Inference System(CANFIS). The SOM is unsupervised and the rest are supervised.

**Figure 3.**
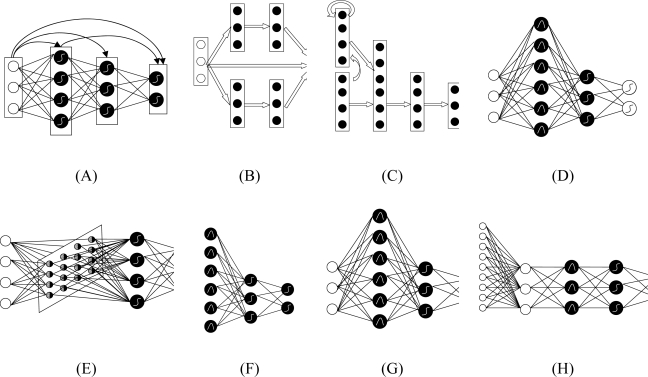
Schematic diagram of eight cloud classifiers: in general the left layer is input layer; the right layer is output layer; and the middle ones are hidden layers. Note that the white circles in the left are input neurons, and in the right are output ones. Black circles are neurons in hidden layer. Lines around circles and arrows between layers show the data flow between neuron and layers respectively. Curves in circles show the transfer function. The linear sum, sigmoid function and Gaussian function are three often used functions. (A) Back Propagation (BP): Its connections can jump over one or more layers. (B) Modular Neural Networks (MNN): It uses several parallel MLPs, and then recombines the results. (C) Jordan-Elman network: It extends the multilayer perceptron with context units, which are processing elements (PEs) that remember past activity. (D) Probabilistic Neural Network (PNN): It uses Gaussian transfer functions and all the weights can be calculated analytically. (E) Self-Organizing Map (SOM): It transforms the input of arbitrary dimension into a one or two dimensional discrete map subject to a topological constraint. (F) Co-Active Neuro-Fuzzy Inference System (CANFIS): It integrates adaptable fuzzy inputs with a modular neural network to rapidly and accurately approximate complex functions. (G) Support Vector Machine (SVM): It uses the kernel Adatron to change inputs to a high-dimensional feature space, and then optimally separates data into their respective classes by isolating those inputs which fall close to the data boundaries. (H) Principal Component Analysis (PCA): It is an unsupervised linear procedure that finds a set of uncorrelated features, principal components, from the input.

In order to compare ANNs with other non-ANN pattern recognition methods, this study selected Principal Component Analysis (PCA) as well, due to its being a cost-effective identifier in terms of time and accuracy in cloud image recognition [30]. In addition, a new mathematical method, Support Vector Machine (SVM) has been compared to evaluate the performances of the different models. SVM is effective in separating sets of data which share complex boundaries and it has been used on GOES-8 and EOS/ MODIS [31,32]. The structure of the eight models can be seen in Figure 3 and their characteristics are described briefly in the following sections.

#### 3.1.1. Brief Description of Cloud Classifiers

1. *Back Propagation (BP):* BP (Figure 3A) is probably the most widely used algorithm for generating classifiers. It is a feed-forward multi-layer neural network [33]. It has two stages: a forward pass and a backward pass. The forward pass involves presenting a sample input to the network and letting activations flow until they reach the output layer. The activation function can be any function. During the backward pass, the network’s actual output (from the forward pass) is compared with the target output and error estimates are computed for the output units. The weights connected to the output units can be adjusted in order to reduce those errors. The error estimates of the output units can be used to derive error estimates for the units in the hidden layers. Finally, errors are propagated back to the connections stemming from the input units.
2. *Modular Neural Networks (MNN):* MNN (Figure 3B) is a special class of Multilayer perceptron (MLP). These networks process their input using several parallel MLPs, and then recombine the results. This tends to create some structure within the topology, which will foster specialization of function in each sub-module.
3. *Jordan-Elman Neural Networks:* Jordan and Elman networks (Figure 3C) extend the multilayer perceptron with context units, which are processing elements (PEs) that remember past activity. In the Elman network, the activity of the first hidden PEs is copied to the context units, while the Jordan network copies the output of the network. Networks which feed the input and the last hidden layer to the context units are also available.
4. *Probabilistic Neural Network (PNN):* PNN (Figure 3D) is nonlinear hybrid networks typically containing a single hidden layer of processing elements (PEs). This layer uses Gaussian transfer functions, rather than the standard Sigmoid functions employed by MLPs. The centers and widths of the Gaussians are set by unsupervised learning rules, and supervised learning is applied to the output layer. All the weights of the PNN can be calculated analytically, and the number of cluster centers is equal to the number of exemplars by definition.
5. *Self-Organizing Map (SOM):* SOM (Figure 3E) transforms the input of arbitrary dimension into one or two dimensional discrete map subject to a topological (neighborhood preserving) constraint. The feature maps are computed using Kohonen unsupervised learning.
6. *Co-Active Neuro-Fuzzy Inference System (CANFIS):* The CANFIS model (Figure 3F) integrates adaptable fuzzy inputs with a modular neural network to rapidly and accurately approximate complex functions. Fuzzy inference systems are also valuable as they combine the explanatory nature of rules (membership functions) with the power of “black box” neural networks.
7. *Support Vector Machine (SVM):* SVM (Figure 3G) has been very popular in the machine learning community for the classification problem. Basically, the SVM technique aims to geometrically separate the training set represented in an *Rn* space, with *n* standing for the number of radiometric and geometric criteria taken into account for classification, using a hyperplane or some more complex surface if necessary. SVM training algorithm finds out the best frontier in order to maximize the margin, defined as a symmetric zone centered on the frontier with no training points included, and to minimize the number of wrong classification occurrences. In order to reach that goal, SVM training algorithm usually implements a Lagrangian minimization technique. It reduces complexity for the detection step. Another advantage is its ability to generate a confidence mark for each pixel classification based on the distance measured in the *Rn* space between the frontier and the point representative of the pixel to be classified: the general rule is that a large distance means a high confidence mark. In this study, the SVM is based upon a training set of pixels with known criteria and classification (cloud/surface). It is implemented using the Kernel Adatron algorithm. The Kernel Adatron maps inputs to a high-dimensional feature space, and then optimally separates data into their respective classes by isolating the inputs which fall close to the data boundaries. Therefore, the Kernel Adatron is especially effective in separating sets of data which share complex boundaries.
8. *Principal Component Analysis (PCA):* PCA (Figure 3H) is a very popular technique for dimensionality reduction. It combines unsupervised and supervised learning in the same topology. In this study, we use PCA to extract principal features of cloud image. These features are integrated into a single module or class. This technique has the ability to identify relatively fewer “features” or components that as a whole represent the full object state and hence are appropriately termed “Principal Components”. Thus, principal components extracted by PCA implicitly represent all the features.

#### 3.1.2. Comparison of Cloud Classifiers

Back Propagation (BP) is the most frequently used ANN network, compared to the other five ANN models. It can approximate any input/output relationships. However, the training of the network is slow and requires lots of training data.

Modular networks such as MLP etc, do not have full interconnectivity between their layers. Therefore, a smaller number of weights are required for the same size network (i.e., the same number of PEs). This tends to speed up training times and reduce the number of required training exemplars. There are many ways to segment a MLP into modules. It is unclear how to design the modular topology best based on the data. There are no guarantees that each module is specializing its training on a unique portion of the data.

Jordan and Elman networks extend the multilayer perceptron with context units. It can extract more information from the data, such as temporal information. Whether it is possible to use Jordan and Elman networks for cloud classification is under discussion.

PNN uses Gaussian transfer functions, its weights can be calculated analytically. It is tend to learn much faster than traditional MLPs.

SOM network’s key advantage is that the clustering produced from it reduces the high-dimensional input spaces into low-dimensional representative features using an unsupervised self-organizing process.

The CANFIS model integrates adaptable fuzzy inputs with a modular neural network. Its classification is usually rapid and accurate. It is suitable for our cloud classification in which Tbb characters are complex.

SVM has been a very popular classification algorithm. It is very common to treat multi-category problems as a series of binary problems in the SVM paradigm. This approach may fail under a variety of circumstances.

PCA is often used directly for principal component and pattern recognition tasks. Nevertheless, PCA is not optimal for separation and recognition of classes.

### 3.2. Evaluation Indices for Model Parameters Screening and Model Testing

The performance of neural networks can be evaluated with three criteria: computational cost (time), training precision, and probability of convergence. The first one can be demonstrated by the training time consumed, and the later two can be indicated by mean square error (MSE), normalized mean square error (NMSE), error (%), correlation coefficient, and accurate rate (%). In addition, AIC (Akaike’s Information Criterion) and MDL (Rissanen’s minimum description length) have been used to evaluate model complexity and accuracy because they are suitable for a large number of samples. These evaluation indexes can be defined as:

#### 3.2.1. Mean square error (MSE):

(1) $$\mathrm{MSE}=\frac{\sum_{j=0}^{p}\;\sum_{i=0}^{N}\;{\left(d_{\mathrm{ij}}-y_{\mathrm{ij}}\right)}^{2}}{N\times P}$$

where *d_ij_* is desired output for exemplar *i* at processing elements *j; y_ij_* is network of output for exemplar i at processing elements *j; N* is number of exemplars in the data set; *P* is number of output processing elements.

#### 3.2.2. Normalized mean square error (NMSE):

(2) $$\mathrm{NMSE}=\frac{P\times N\times \mathrm{MSE}}{\sum_{j=0}^{p}\;\frac{N\sum_{i=0}^{N}\;d_{\mathrm{ij}}^{2}-{\left(\sum_{i=0}^{N}\;d_{\mathrm{ij}}\right)}^{2}}{N}}$$

where *dy_ij_* is denormalized network of output for exemplar *i* at processing elements j; *dd_ij_* is denormalized desired output for exemplar *i* at processing elements *j.*

#### 3.2.3. Percent Error (%):

(3) $$\mathrm{Error}=\frac{100\times \sum_{j=0}^{p}\;\sum_{i=0}^{N}\;\frac{\left|{\mathrm{dy}}_{\mathrm{ij}}-{\mathrm{dd}}_{\mathrm{ij}}\right|}{{\mathrm{dd}}_{\mathrm{ij}}}}{N\times P}$$

#### 3.2.4. Correlation coefficient (Corr) :

(4) $$r=\frac{\sum_{j=0}^{p}\;\sum_{i=0}^{N}\;\left(d_{\mathrm{ij}}-\bar{d}\right)\left(y_{\mathrm{ij}}-\bar{y}\right)}{\sqrt{\sum_{j=0}^{p}\;\sum_{i=0}^{N}\;{\left(d_{\mathrm{ij}}-\bar{d}\right)}^{2}\;\sum_{j=0}^{p}\;\sum_{i=0}^{N}\;{\left(y_{\mathrm{ij}}-\bar{y}\right)}^{2}}}$$

where *d̄* is desired output for exemplar; *ȳ* is network of output for exemplar.

#### 3.2.5. Accuracy rate (%)

$$\mathrm{Accuracy rate}=\frac{n}{N}\times 100\%$$

where *n* is the number of samples which have been detected correctly by classifier.

#### 3.2.6. Akaike’s Information Criterion (AIC):

(5) AIC(k)=N ln(MSE)+2k

where *k* is the number of network weights.

#### 3.2.7. Rissanen’s Minimum Description Length (MDL):

(6) MDL(k)=N ln(MSE)+0.5k ln(N)

According to Equations (1) through (6), a model with high precision has less MSE, NMSE, error (%), and correlation coefficient, and the AIC and MDL for simpler cloud classifiers are also less.

### 3.3. Cloud Classifier Parameters

There is no unified method to determine optimal parameters at present. Therefore, classifier parameter such as the number of hidden layers and neurons, learning rules and so on, are greatly dependent on experience and numerous tests. Parameters of the cloud classifiers in this study are listed in Table 4.

### 3.4. Training and Validation of Cloud Classifier

To learn the relation between the input and output vectors, this study trained the ANN classifier with 80% of meteorologists’ manually selected samples (2,290). Connection weights were adjusted to minimize the root-mean-square error between the desired output and the estimates from classifiers. The ability of ANN to represent highly nonlinear relationships highlights an important caution for the training of neural networks: they can be over trained. Therefore, we choose 10% of training samples (229) randomly for cross-examination to identify the appropriate training interval. In order to analyze the capability of classifier, this study validates cloud classifier at two levels: pixel level and image level. As for the former one, the remaining 20% of samples (574) were used.

The GY-2C geostationary satellite provides cloud images at intercontinental coverage at relative high temporal resolution. Therefore, it requires evaluation of the classification results at intercontinental scale and also in daytime, night and twilight. However, because of the different satellite configuration and complexity of cloud dynamics, it is not proper to use cloud classification result from other satellite as the “truth”. Likewise, ground-based cloud assessment is not suitable for the validation either, because it observes clouds from the bottom while satellites do it from the top. Therefore, this study invited several experienced meteorological experts to evaluate the results of the cloud classification based on analyzing the false RGB images from the FY-2C multi-channel data with their experience and knowledge. This study also demonstrated the capability of ANN classifier by comparing it with the clustering method currently used by FY-2C operational products both at pixel level and cloud patch level.

## 4. Result and Analysis

In subsection 4.1, pixel level cloud classification results from the eight different models are presented for cross-examination (training) and validation (test). In subsection 4.2, cloud classification images are analyzed by comparing with the current operational FY-2C cloud classification products based on three typical challenging cases in daytime, night, and twilight, respectively.

### 4.1. Pixel-level Evaluation of Classification Results

**Figure 4.**
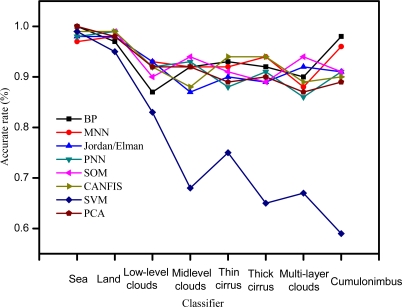
The accuracy rate of the eight cloud classifiers for the test data.

The result of training cross-examination and test validation of the eight cloud classifier are presented in Table 5 and Figure 4. It shows that the training time for all methods is less than half minute with the exception of SVM. More detailed analyses are described in the following section.

#### 4.1.1. Cross-Examination Results of Classification

Almost all the methods work well except the SVM. The MSE, NMSE of top seven models are not more than 0.03, 0.05 respectively and model errors are less than 10% and correlation coefficient are more than 0.98. Overall, the performance of cloud classifiers has the following decreasing order: Jordan/Elman, PNN, SOM, BP, MNN, CANFIS, PCA, and SVM. As for the complexity of the model, AIC and MDL of all the studied cloud classifiers are not more than 1,000 except PNN and the SVM. Their complexity has the following increasing tendency: BP, MNN, Jordan/Elman, PCA < CANFIS, SOM < PNN < SVM.

#### 4.1.2 Test Results of Classification

As shown the validation of test results in Table 5 and the Figure 4, almost all methods have good accuracy, except SVM. The precision of the classifiers ranks at the following order: PNN, SOM > BP, MNN, Jordan/Elman > CANFIS, PCA > SVM. Their MSE, NMSE, and errors are not more than 0.02, 0.03, 10%, respectively, and correlation correlation (corr) reaches 0.99. As for the complexity of the model, AIC and MDL of all models are not more than 1,000, except for SVM. The simplicity has the following tendency: PNN, PCA, SOM > BP, MNN, Jordan/Elman > CANFIS > SVM. Therefore, validation of the test results shows that all methods perform consistently with that of cross-examination (training) results.

In conclusion, performance evaluation of the eight classifiers both in training and testing indicates that all the six ANNs outperform non-ANN methods (slightly better than PCA and much better than SVM). Among the ANNs, the SOM and PNN show best results in cloud classification compared to other four ANNs (BP, MNN, Jordan/Elman, CANFIS) in terms of model precision and simplicity. However, the advantages are not very prominent and all these six ANN models can literally obtain acceptable results, as long as trained by sufficient samples.

The result also shows that the method support vector machine (SVM) is not applicable for FY-2C cloud classification, with error near 50% in both training and testing period. The reason may be that SVM treats multi-category problems as a series of binary problems and it fails to capture the high variability of the cloud system dynamics.

#### 4.1.3. Comparison with FY-2C Operational Cloud Classification Products

In order to compare the classification result of proposed ANNs with the clustering method currently used for FY-2C operational products, this study chose the SOM model as a representative of ANN classifiers. The remaining 20% testing samples are used for the comparison since they are the validation data used for the eight ANN cloud classifiers.

Because of the slight difference on operational product and ANN model definition of cloud classes, this study chooses only their common types for comparison: sea, thick cirrus, cumulonimbus, and land. Comparison results are listed in Table 6. They show that the ANN-based cloud classification result has been improved greatly from the clustering method. ANN model can detect non-cloud (clear land and clear sea) with accurate rate about 99% (99.01% for sea and 98.51 for land), and 88.79% for thick cirrus, while less than 85% (83.02% for sea and 48.35% for land) and 26.14% for thick cirrus for the FY-2C operational product, respectively.

### 4.2. Cloud path-Level Evaluation of Classification Results

Several images at 07: 00 UTC, 15: 00 UTC, 23: 00 UTC on 9 July2007 are used here to analyze the ANN capabilities on cloud classification. Those images cover cloud samples for daytime, night, and twilight. The main reason to validate the classification results at the three UTC time stamps is because of the fact that they represent the typical challenging cases for cloud classification. Our purpose here is to provide a quantitative way to compare and analyze the capabilities of ANN-based cloud classification methods not to make general conclusions based on several cases.

#### 4.2.1. Case 1: high latitude case

**Figure 6.**
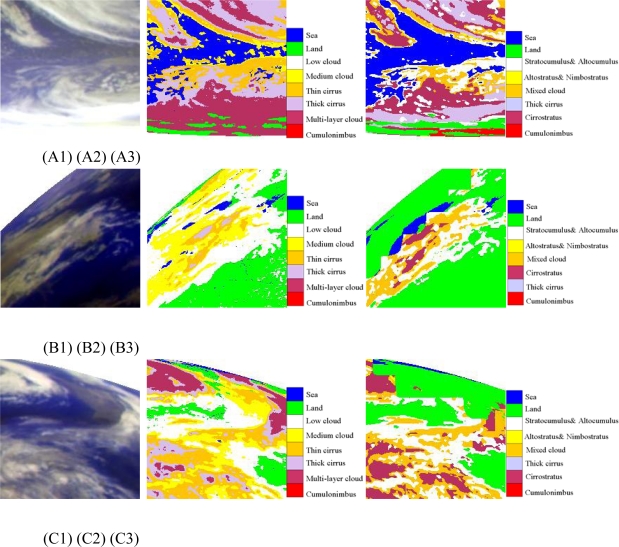
(A) The first line (A1, A2, A3) are high latitude cases at 07: 00 UTC (daytime); (B) The second line (B1, B2, B3) are high latitude cases at 15: 00 UTC (night); (C) the third line (C1, C2, C3) are high latitude cases at 23: 00 UTC (twilight); The first column (A1, B1, C1) are false RGB composite of Tbb of IR1, IR2 and WV; The second column (A2, B2, C2) are cloud classification results of ANN; The third column (A3, B3, C3) are results of FY-2C operational products.

Cloud/surface classification in high latitude areas is challenging because Tbb of land is low, which is close to that of clouds [34]. Therefore, this study has chosen three cases (case A, B, C) in high latitude as shown in Figure 6.

As for case A, the FY-2C operational product (A3) misclassified the cloud by treating thick cirrus as cumulonimbus in the lower part of picture because of their similar brightness temperature in IR and WV channels. However, for the study area, it is not possible to have cumulonimbus characterized by strong convection. ANN model (A2), which uses more channels and features, determines that the region is covered mainly by low-level and midlevel clouds. It conforms to its local meteorological condition.

As for cases B and C, It shows that the current FY-2C cloud classification products (B3 and C3) usually have obvious quadrate edge due to its 32-pixel × 32-pixel window-based clustering method, while that of ANN model (B2 and C2) has smooth boundary and its configuration is close to the reality.

#### 4.2.2. Case 2: Cumulonimbus case

**Figure 7.**
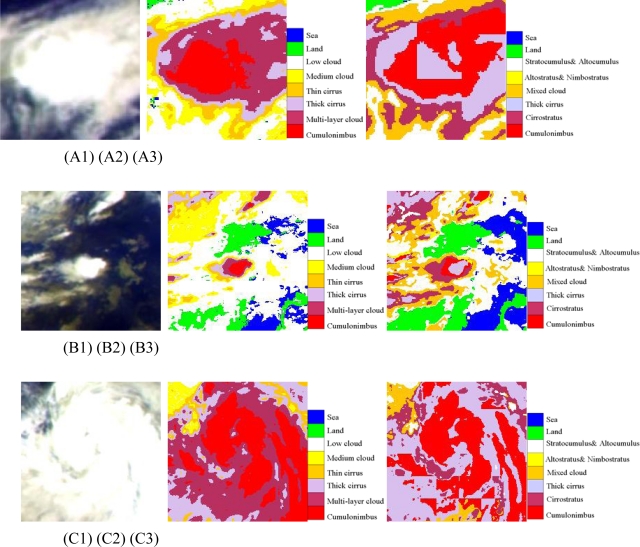
(A) The first line (A1, A2, A3) are cumulonimbus cases at 07: 00 UTC (daytime); (B) The second line (B1, B2, B3) are cumulonimbus(Cb) cases at 15: 00 UTC (night); (C) the third line (C1, C2, C3) are cumulonimbus(Cb) cases at 23: 00 UTC (twilight); The first column (A1, B1, C1) are pseudo-color composite map of Tbb of IR1, IR2 and WV; The second column (A2, B2, C2) are cloud classification results of ANN; The third column (A3, B3, C3) are results of FY-2C operational products.

Strong convective precipitation is closely related to the characters of cumulonimbus, one typical strong convection cloud. Therefore the study of configuration and texture of cumulonimbus (Cb) has been of great interest for meteorologists. This study examined three cases (case A, B and C) in tropical zone and temperate zone as shown in Figure 7.

For case A and C, cumulonimbus (Cb) identified by ANN (A2 and C2) are spherical napiform and more continuous compared to FY-2C operational model (A3 and C3). The size of cumulonimbus (Cb) from FY-2C operational model in case A are larger than those of ANN as it misjudged most of cirrus carpet (thick cirrus) as cumulonimbus. As for the case B, cumulonimbus (Cb) of ANN model (B2) is bounded by multi-layer clouds, thick cirrus and thin cirrus respectively, while there is only thick cirrus at the center of the cloud patch for FY-2C operational model (B3). Cumulonimbus (Cb) is outside of it. The result of ANN model is more consistent with reality while the current FY-2C operational product misjudged cloud types in this case.

#### 4.2.3. Case 3: Cirrus case

**Figure 8.**
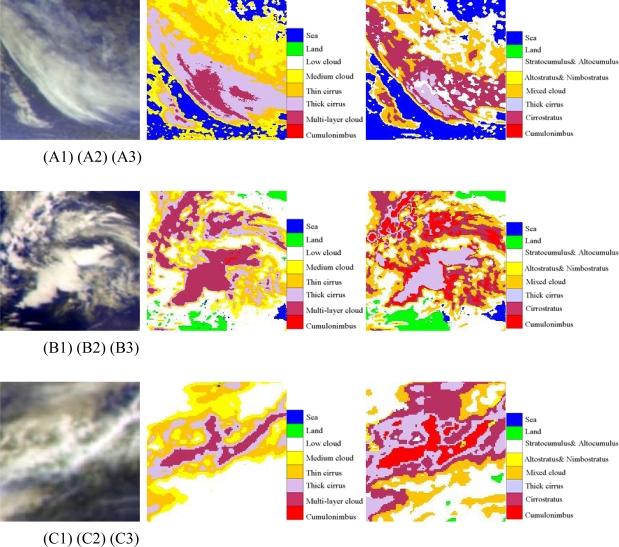
(A) The first line (A1, A2, A3) are cirrus at 07: 00 UTC(daytime); (B) The second line (B1, B2, B3) are cirrus cases at 15: 00 UTC (night); (C) the third line (C1, C2, C3) are cirrus cases at 23: 00 UTC (twilight); The first column (A1, B1, C1) are pseudo-color composite map of Tbb of IR1, IR2 and WV; The second column (A2, B2, C2) are cloud classification results of ANN; The third column (A3, B3, C3) are results of the FY-2C operational product.

Considering the impact of thin cirrus and thick cirrus on solar radiation quite different, this study categorized cirrus clouds into thick one and thin one and establishes corresponding dataset. To analyze the classification results of cirrus, this study has chosen three cases: case A, B and C as shown Figure 8.

As for the case A, it shows that the ANN model is able to identify most of the thin cirrus from medium clouds, while the FY-2C operational product treats them as altostratus or nimbostratus. The reason is that ANN model established a dataset containing thin cirrus with multi-channels data, and FY-2C operational product adopts clustering method and only uses two channels data (IR1 and WV).

As for the case B and C, it shows that the ANN model is able to identify most thick cirrus well, while FY-2C operational products misjudge multi-layer clouds and thick cirrus as cumulonimbus (Cb). Therefore, ANN classifier found to be better in distinguishing cirrus than FY-2C operational product.

## 5. Conclusions and Discussion

This study analyzed six ANN cloud classification methods and compared them with the clustering method adopted by the current FY-2C operational product, and two other typical pattern recognition methods such as PCA and SVM. Results of this study demonstrated that given sufficient training samples, both ANN models and PCA can obtain relatively better classification results (with less than 10% classification error), as opposed to the SVM results. Cloud classification results of ANN models work slightly better than the traditional method, PCA. Among the six ANN methods, this study found that SOM and PNN work best than other four ANN methods (i.e., BP, MNN, Jordan/Elman, and CANFIS).

Compared to the current FY-2C cloud classification product, ANN classifier not only improved the accuracy at the pixel level but also on the cloud patch image level. Several cases of cumulonimbus, cirrus and clouds in high latitude and in different daytime, night and twilight demonstrate that ANN classifier outperforms the existing window-based clustering method. It is desirable for us to upgrade the current FY-2C cloud classification method with one of top ANN performer (i.e., SOM).

However, in reality the ANN classifier might not be able to achieve such good results as demonstrated in this study. There are mainly two reasons to constrain the performance of the ANN models. First, cloud top brightness temperatures vary greatly in different seasons, while cloud samples of this study were only manually collected in one summer season. To truly establish an automated cloud classification algorithm for the FY-2C satellite, more work needs to be carried out to analyze whether it is possible to build a cloud classification method independent of regions and seasons, given that “sufficient” training samples can be collected. Second, due to its relatively coarse resolution, geostationary meteorological satellite is not able to capture the spatial variation of clouds as well as compared to some polar-orbiting satellites, especially for smaller cloud systems. With the development of future sensors and the combination of geostationary satellites and polar-orbiting satellites, it will provide us better remote sensing cloud imagery for cloud classifications than the current ones.

## Figures and Tables

**Figure 5.**
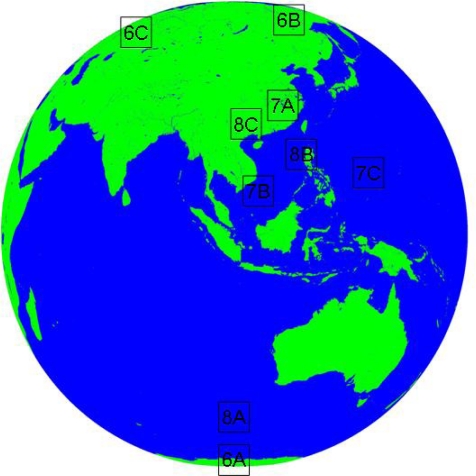
Location of cases: 6X (6A, 6B,6C) is the location of case A, B, C in high latitude (Figure 6); 7X (7A, 7B,7C) is the location of case A, B, C of Cumulonimbus (Figure 7); and 8X (8A, 8B, 8C) is the location of case A, B, C of cirrus (Figure 8).

**Table 1.** Specifications of VISSR channels: spectral range and spatial resolutions.

**Channel No.**	**Channel name**	**Spectral range (μm)**	**Spatial resolution (km)**
1	IR1	10.3–11.3	5
2	IR2	11.5–12.5	5
3	IR3(WV)	6.3–7.6	5
4	IR4	3.5–4.0	5
5	VIS	0.55–0.90	1.25

**Table 2.** The set of classes and samples in this study.

**Classes**	**Samples**	**Description**
**Sea**	184	Clear sea
**Land**	266	Clear land
**Low-level clouds**	405	Stratocumulus (Sc), Cumulus (Cu), Stratus (St), Fog, and Fractostratus (Fs)
**Midlevel clouds**	379	Altocumulus (Ac), Altostratus (As), and Towering Cumulus
**Thin cirrus**	415	Thin cirrus
**Thick cirrus**	440	Thick cirrus
**Multi-layer clouds**	371	Cumulus congestus (Cu con), Cirrostratus (Cs) and Cirrocumulus (Cc)
**Cumulonimbus**	404	Cumulonimbus(Cb)
**Sum**	2864	

**Table 3.** Selected Features according to the Gray Level Co-occurrence Matrices (GLCM) for cloud classification. Note that Ti (T1, T2, T3) is the Tbb of channel i (IR1, IR2 and WV) and Gi (G1, G2, G3) is the gray value of channel i (IR1, IR2 and WV).

**Features**	**Parameters**	**Description**
Spectral features	T1,T2, T3	Top brightness temperature of IR1,IR2,WV
Gray features	G1, G2, G3	Gray value of IR1,IR2,WV
	G1–G2, G1–G3, G2–G3	
Assemblage features	T1–T2, T1–T3, T2–T3	The combination of infra split window and water vapor channel
	(G1–G2)/G1, (G1–G3)/G1, (G2–G3)/G2	

**Table 4.** Parameters of eight cloud classifiers*.

**Type of network**		**Output layer**	**Hidden layer**		

		**Learning step**	**Number of hidden layer**	**Number of Neurons**	**Learning step**
**ANN**	**BP**	0.10	2	9,4**^(1)^	0.10
	**MNN**	0.10	1	4,4**^(2)^	0.10
	Jordan/Elman***^(1)^	0.10	1	9	0.10
	PNN***^(2)^	1.00	1	6	1.00
	SOM Network***^(3)^	0.10	1	9	1.00
	CANFIS***^(4)^	0.10	1	4	0.10

**SVM**		0.01			

PCA***^(5)^		0.10			

* Learning momentums of 6 ANN models are sets as 0.7, and TanhAxons are used as transfer functions. The learning momentums and TanhAxons are the same for SVM and PCA in output layers. The activation function for TanhAxon is that: *f*(*x_i_*,*w_i_*) = tanh(*x_i_^lin^*), where *x_i_^lin^* = *βx_i_* is the scaled and offset activity inherited from the LinearAxon. More information concerning classifiers’ parameters can be found at *http://www.neurosolutions.com/downloads/documentation.html.*

** Number of Neurons: (1): Hidden Layer1:9; Hidden Layer 2:4; (2): Upper PEs = 4; Low PEs = 4.

*** Some structure parameters of models: (1) Time:0.4; Integrator Axon; (2): Cluster: 75; Competitive: conscience; Metric: Euclidean;(3) Rows:5; columns:5; Starting:2; Final radius: 0; Neighborhood shape: Square Kononen Full; (4) Gamma axon memory; Depth in: 10; Trajectory:50; (5) Learning rule: Sanger full; Principal 4.

**Table 5.** The evaluation result of the eight cloud classifiers for the training and testing data.

		**Cross-examination (Training)**						**Test**					
**Method**	**Time(S)**	**MSE**	**NMSE**	**Corr**	**Errol (%)**	**AIC**	**MDL**	**MSE**	**NMSE**	**Corr**	**Errol (%)**	**AIC**	**MDL**
**BP**	30.00	0.01	0.02	0.99	9.35	−399.35	−214.35	0.01	0.02	0.99	8.87	−2693.14	−3376.66
**MNN**	21.00	0.02	0.05	0.98	9.20	−106.53	−46.16	0.01	0.03	0.99	8.87	−2832.60	−2599.99
**Jordan/Elman**	22.00	0.01	0.03	0.99	8.75	−31.27	−126.34	0.02	0.03	0.99	8.88	−3852.71	−3398.99
**PNN**	63.00	0.01	0.03	0.99	8.52	2812.18	3428.47	0.01	0.02	0.99	7.75	−20.61	2160.85
**SOM**	21.00	0.03	0.05	0.98	8.92	854.12	947.30	0.01	0.02	0.99	7.74	−1340.14	−699.23
**CANFIS**	44.00	0.02	0.04	0.98	10.53	464.56	924.43	0.02	0.03	0.99	9.23	−4851.31	−3776.72
**SVM**	22.30	0.67	2.01	−0.08	49.82	38837.55	4820.26	0.67	2.01	−0.08	49.82	38837.55	48200.26
**PCA**	19.00	0.02	0.03	0.99	10.42	−46.33	−48.75	0.01	0.02	0.99	10.18	−1449.86	−1293.18

**Table 6.** Accuracy rate of FY-2C operational product and ANN model (%).

**Type**	**FY2C product**	**ANN cloud classification**
Sea	83.02	99.01
Thick Cirrus	26.14	88.79
Cumulonimbus	76.49	90.74
Land	48.35	98.51
